# Quality of Life of Women with Polycystic Ovary Syndrome

**DOI:** 10.3390/medicina60020294

**Published:** 2024-02-09

**Authors:** Natalia Ligocka, Karolina Chmaj-Wierzchowska, Katarzyna Wszołek, Maciej Wilczak, Katarzyna Tomczyk

**Affiliations:** Department of Maternal and Child Health, Poznan University of Medical Sciences, 60-701 Poznan, Poland

**Keywords:** polycystic ovary syndrome, menstrual disorders, infertility, hirsutism, insulin resistance, quality of life

## Abstract

*Background and Objectives*: Polycystic ovary syndrome (PCOS) is an endocrine disorder characterized by multiple hormonal and metabolic abnormalities, including insulin resistance, hyperandrogenism, and disturbances in lipid and carbohydrate metabolism. The objective of this study is to assess the quality of life of women diagnosed with polycystic ovary syndrome (PCOS) and to identify any factors within the study group that may impact the scores related to quality of life. *Materials and Methods*: This research was carried out among women diagnosed with PCOS. An original questionnaire, developed through an online Google Forms survey, was utilized as the research instrument and distributed through social networks and support groups to women facing PCOS. This study encompassed a participant pool of 200 women with PCOS, aged 24 years or older. For the analytical component, Pearson’s *χ*^2^ test was employed—a nonparametric test designed to assess the relationship between two variables measured on a qualitative scale. The chosen level of statistical significance was set at *p* < 0.05. *Results*: The analysis revealed that the quality of life of the women under study was not linked to the duration of the disease or comorbidities. However, a significant association was observed with the inconvenience caused by PCOS symptoms. Women experiencing very bothersome symptoms of PCOS reported a lower quality of life compared to those with symptoms rated as not very bothersome. Despite the majority of women with PCOS rating their quality of life as good or very good, they often find the associated symptoms of PCOS bothersome. Women reporting lower quality of life tend to acknowledge the impact of PCOS on their lives, experience a sense of lack of control over the disease, struggle with depression, and do not accept their physical appearance. *Conclusions*: Hence, the support from specialists like endocrinologists, gynecologists, and nutritionists becomes crucial for many women dealing with PCOS. Adopting a healthy lifestyle, incorporating a balanced diet, and engaging in regular physical activity can assist in managing the troublesome symptoms of PCOS, thereby enhancing overall quality of life. In instances of emotional difficulties, seeking psychological support is equally important, and the significance of support and acceptance from loved ones should not be overlooked.

## 1. Introduction

Polycystic ovary syndrome (PCOS) is an endocrine disorder characterized by multiple hormonal and metabolic abnormalities, including insulin resistance, hyperandrogenism, and disturbances in lipid and carbohydrate metabolism [1,2,3,4,5,6,7]. Dysbiosis of gut microbiota may play a pathogenic role in the development of PCOS [8]. Several criteria exist for diagnosing PCOS, such as the National Institutes of Health (NIH) criteria, Androgen Excess Society (AES) criteria, and Rotterdam criteria. Among these, the Rotterdam criteria are the most widely utilized [9,10,11,12]. To receive a diagnosis of PCOS, a minimum of two of the three Rotterdam criteria must be satisfied: ultrasound evidence of polycystic ovaries (presence of at least 20 Graff follicles measuring 2–9 mm in diameter and/or ovarian volume >10 mL), infrequent ovulation or absence of ovulation, and clinical and/or biochemical indications of hyperandrogenism [13]. When employing these criteria for PCOS diagnosis, it is essential to simultaneously rule out other endocrine causes [13]. Women grappling with PCOS frequently contend with symptoms that can be personally challenging. Therefore, the paramount approach involves providing them with support and understanding, creating a sense of safety. This approach not only facilitates effective interviews but also contributes to formulating a treatment plan geared toward mitigating or addressing the prevailing symptoms and achieving the desired outcomes for the patient. Consequently, it is essential to delve into the quality of life experienced by women with PCOS and the prevalent challenges they encounter [14,15,16,17,18,19,20]. This includes exploring aspects such as family life, social interactions, sexual well-being, professional engagements, parenthood, fertility treatments, and potential feelings of helplessness linked to issues like hirsutism or accompanying metabolic conditions.

The objective of this study was to conduct a self-assessment of the quality of life of women with PCOS and to analyze the factors influencing the aforementioned quality of life scores.

## 2. Materials and Methods

The research was carried out among women diagnosed with PCOS. An original questionnaire entitled “Assessment of the quality of life of women with polycystic ovary syndrome”, developed through an online Google Forms survey, was utilized as the research instrument and distributed through social networks and support groups to women with verified PCOS. The study included a group of 200 participants with PCOS.

The questionnaire consisted of 28 questions (including 4 multiple-choice ones). The first part of the questionnaire had questions related to general demographic information (age, place of residence, marital status, education, professional status, and socioeconomic conditions). The next questions concerned PCOS (when was the disease diagnosed, what are the symptoms and other diseases, and what is the treatment used) and fertility. The third part of the survey included questions regarding the assessment of quality of life and experienced emotions (depression/anxiety/sadness, a feeling of lack of control or acceptance of physical appearance, and support from family).

Data analysis was conducted using TIBCO Software Inc. (2017) Statistica, version 13, and Microsoft Excel, version 2019 (Microsoft Office). The analysis of the gathered data took the form of both descriptive and analytical statistics. Descriptive statistics were conducted through a Microsoft Excel spreadsheet, which included the creation of graphs based on the collected data. For the analytical component, Pearson’s *χ*^2^ test was employed—a nonparametric test designed to assess the relationship between two variables measured on a qualitative scale. The chosen level of statistical significance was set at *p* < 0.05.

## 3. Results

The study cohort comprised 200 women diagnosed with PCOS, aged 24 years or older.

### 3.1. Characteristics of the Study Group of Women with Polycystic Ovary Syndrome

The predominant segment of the study group consisted of women aged 24 to 29, making up 53% of the participants. Geographically, 34.5% resided in large cities with populations exceeding 100,000, while 32% lived in rural areas. Among the participants, 41% were married. In terms of education, 60% of the women held higher education degrees, and a majority, accounting for 71%, were economically active. Assessing socioeconomic conditions, 57% rated them as good (see Table 1). Within the study group, 18% of the women had children, while the remaining 82% did not. Among those attempting to conceive, 55% made no efforts, 41.5% encountered difficulties, and 3.5% reported no problems. For women with children, the duration of attempts to conceive varied, with 38.6% lasting 1–2 years, 31.3% up to a year, 21.7% spanning 3–5 years, and 8.4% extending beyond 5 years. The manifestation of PCOS primarily presented as irregular menstrual cycles (80.5%) and the characteristic ultrasound image of polycystic ovaries (71.5%). Other common manifestations included infrequent or absent ovulation (61%), insulin resistance/hyperinsulinemia (60.5%), hirsutism (50%), overweight/obesity (47%), difficulties in getting pregnant (45%), acne (44%), and bloating (41.5%). Less frequently reported symptoms included amenorrhea (32%), hyperandrogenism (26.5%), infertility (24%), and androgenetic alopecia (17.5%). Regarding comorbidities, 30.5% of women had hypothyroidism/Hashimoto’s disease, 9% had lipid disorders, 7.5% experienced hypertension, 5.5% had diabetes, and 1.5% had hyperthyroidism.

The women surveyed typically rated the severity of their symptoms as bothersome (40.5%), moderately bothersome (26.5%), or very bothersome (24%). Less frequently, respondents rated their symptoms associated with PCOS as not very bothersome (8.5%) or not bothersome at all (0.5%). Of the women surveyed, 79% were undergoing treatment for PCOS-related symptoms, while 21% were not receiving treatment for PCOS. A total of 96% of the surveyed women were under the regular care of a gynecologist, and 50.5% were under the regular care of an endocrinologist. A smaller percentage of women surveyed were under the regular care of a nutritionist (13%), psychologist (11%), dermatologist (8.5%), psychiatrist (8%), or diabetologist (7.5%) (see Table 2).

### 3.2. Self-Assessment of Quality of Life of Women with Polycystic Ovary Syndrome

The women who participated in the survey generally assessed their quality of life as good (self-assessment of quality of life), rated by 61.5%. Following this, 20% rated their quality of life as very good, while 15% indicated a satisfactory quality of life. A small percentage of respondents, 2.5%, rated their quality of life poorly, and 1% rated it very poorly. The presence of PCOS affected “some sphere” of life for 84% of the surveyed women, whereas 16% acknowledged that PCOS had no impact on their lives. Within the study group, PCOS predominantly affected the sexual sphere for 79.2% and, to a lesser extent, the family (61.9%), social (50.6%), and occupational (24.4%) spheres. Sadness attributed to PCOS was reported by 79% of the surveyed women, with only 21% indicating that they experienced no sadness or anxiety due to PCOS. A feeling of being out of control due to PCOS was experienced by 75% of the women, and 26% struggled with depression. Acceptance of their physical appearance was noted by only 40.5% of the surveyed women, while 59.5% did not accept their appearance. Furthermore, 67.5% of the women admitted to having low self-esteem due to the presence of PCOS, with 32.5% reporting no perceived impact. Regarding family support, the surveyed women typically rated it as medium (43%) or good (32%), with fewer respondents rating it as very good (14%), bad (9%), or very bad (2%) (see Table 3).

### 3.3. Assessment of Factors Affecting the Quality of Life of Women with Polycystic Ovary Syndrome

The analysis revealed that the quality of life of the women under study was not linked to the duration of the disease (*χ*^2^ = 8.98; *p* = 0.344) or comorbidities (*χ*^2^ = 4.04; *p* = 0.401). However, a significant association was observed with the inconvenience caused by PCOS symptoms (*χ*^2^ = 22.25; *p* < 0.01). Women experiencing very bothersome symptoms of PCOS reported a lower quality of life compared to those with symptoms rated as not very bothersome (refer to Table 4).

Likewise, the correlation between the assessment of quality of life and factors such as treatment, adherence to diet, and physical activity was investigated through Pearson’s *χ*^2^ test analyses. The outcomes of these analyses are presented in Table 5, but they proved to be statistically insignificant (*p* > 0.05). Only the connection between quality of life and dietary adherence approached statistical significance (*χ*^2^ = 5.67; *p* = 0.059). Women following a diet tended to give lower ratings of their quality of life.

Additionally, through Pearson’s *χ*^2^ test analyses, the relationship between the assessment of quality of life and factors such as having children and experiencing negative emotions was explored. The results of these analyses are detailed in Table 6. No significant association was found between quality of life and having children (*χ*^2^ = 1.66; *p* = 0.436) or experiencing sadness (*χ*^2^ = 3.40; *p* = 0.183). However, quality of life was observed to be linked with the impact of the disease on one’s life (*χ*^2^ = 13.14; *p* < 0.01), feeling out of control (*χ*^2^ = 10.17; *p* < 0.01), and depression (*χ*^2^ = 19.16; *p* < 0.001). Women who acknowledged that PCOS had an impact on their lives, those feeling out of control due to the disease, and those struggling with depression reported lower quality of life.

This study also investigated whether the quality of life of women with PCOS was linked to the impact of the disease on self-esteem. To explore this, a series of analyses were conducted using Pearson’s *χ*^2^ test, and the results, detailed in Table 7, were found to be statistically significant (*p* < 0.05). The findings indicated that women who did not accept their physical appearance, had low self-esteem, and lacked family support tended to rate their quality of life as lower.

## 4. Discussion

PCOS stands as the most prevalent endocrine disorder among women of reproductive age. The range of clinical manifestations associated with PCOS are substantial, encompassing hormonal, metabolic, lipid, and carbohydrate metabolism disorders [16]. These conditions collectively exert a significant impact on the quality of life for affected women [1]. Given the diverse array of clinical symptoms and the associated dependencies on the occurrence of additional diseases, it is imperative to approach each patient with PCOS on an individual basis. Tailoring the treatment to encompass the patient’s symptoms, concurrent conditions, and expectations for therapeutic outcomes is crucial for quality of life. Women with PCOS suffer more from depression and sexual dysfunction than healthy patients [21,22]. In our study, the majority of women in the sample rated their quality of life (self-assessment of quality of life) as good, accounting for 61.5%, while 20% of respondents perceived themselves as having a very good quality of life. Unfortunately, a significant portion, 79% of the surveyed women, experienced feelings of sadness related to the disease. Additionally, 75% declared a lack of control over PCOS, and 26% of the women reported struggling with depression. Moreover, 67.5% of the surveyed women admitted to experiencing low self-esteem due to the presence of PCOS, with only 40.5% accepting their physical appearance.

Women dealing with PCOS contend with various health challenges that manifest to varying degrees across different aspects of their lives. The predominant symptoms of PCOS include irregular menstrual cycles [23] and hirsutism [16]. Sidra et al. [24] noted that irregular menstrual cycles (71.8%) and hirsutism (68.7%) were the most commonly mentioned symptoms by women with PCOS [24]. Hirsutism, identified by Bazarganipour et al. [25], is frequently reported by women with PCOS, with Mohammad et al. [16] stating that it affects nearly 70% of women, often impacting their sense of femininity [16]. Rzońca et al. [26] reported that the longer the duration of the PCOS, the lower the overall quality of life of affected women, affecting various spheres such as social, physical, and environmental well-being [26]. Conversely, Stańczyk et al. [27] observed a prevalence of lowered self-esteem among most women with PCOS, particularly those grappling with hirsutism and lacking robust social support [27]. Contrary to some previous findings, our study did not establish a correlation between the duration of illness or the presence of additional diseases and the quality of life. However, it did reveal a noteworthy association between the severity of PCOS symptoms and the overall quality of life. Women with highly bothersome PCOS symptoms tended to rate their quality of life as lower.

Struggling with issues such as excessive body hair, challenging-to-control acne, increased hair loss, or heightened sebum production can significantly diminish a woman’s self-esteem, particularly in terms of her physical attractiveness, leading to embarrassment and potentially contributing to the development of psychosocial disorders [1,2]. Additionally, conditions like overweight, obesity, insulin resistance, and hyperinsulinemia can either be a cause or consequence of metabolic disorders, substantially impacting the deterioration of quality of life and self-perception [28,29]. For women with PCOS, excessive body weight and the prolonged effort to shed it can also negatively affect their self-esteem [27,30]. A study by Moghadam et al. [20] highlighted obesity as a significant contributor to poor quality of life, particularly due to its association with negative psychological symptoms [20]. In addressing most symptoms linked to PCOS, lifestyle modification is often recommended. This involves adopting a diet that supports weight maintenance or reduction, alongside regular exercise—considered the primary nonpharmacological treatment for women with PCOS. Lifestyle modifications yield substantial improvements in metabolism, reproductive function, and a reduction in insulin resistance [17,31]. A reduction in body weight has been found to primarily enhance insulin sensitivity in target tissues. This weight loss leads to increased sex hormone binding globulin (SHBG) and decreased testosterone levels, thereby alleviating symptoms of hyperandrogenism. This mechanism also contributes to rectifying endocrine dysfunction, regulating menstrual cycles, and enhancing ovulation frequency, thereby improving fertility [32,33,34]. In the context of lifestyle modification, maintaining an appropriate diet is crucial. This involves primarily reducing the intake of high glycemic index carbohydrates, adopting a low-calorie diet, increasing fiber and polyunsaturated fat consumption, and decreasing saturated fats [3,18,35,36]. Another vital aspect of modifying the lifestyle of women with PCOS is regular physical exercise, which, when combined with an appropriate diet, can lead to a reduction in excessive body weight and alleviate bothersome PCOS-related symptoms, ultimately contributing to enhanced well-being. Regular physical activity is recommended at least three times a week, with each session lasting at least 30 min. The selection of suitable physical activities depends on individual needs and preferences, ensuring alignment with a woman’s current capabilities [29]. Although our study did not confirm a direct correlation between physical activity and quality of life, it did highlight that women who did not accept their physical appearance, had low self-esteem, and lacked family support tended to rate their quality of life as lower. Intriguingly, there was a noteworthy trend in quality of life among those adhering to dietary recommendations, approaching statistical significance. Specifically, women on a diet tended to rate their quality of life more negatively.

Women with PCOS often face challenges in conceiving or may be diagnosed with infertility. This contributes to a decline in the quality of life for these women, as the desire to have a child holds significant importance in the lives of a certain proportion of women. It represents the realization of their dreams and a strengthening of family ties with their partners. According to Sulaiman et al. [29], infertility affects 38.4% of women with PCOS [29]. Additionally, Barnard et al. [23] associate PCOS with elevated levels of depression [23]. Sidra et al. [24] share a similar perspective, reporting an increasing incidence of depression among women with PCOS, with a result of 61.8% [24]. In our study, a slightly different result was obtained, with 26% of surveyed women confirming the presence of depression. Given the gravity of depression in the context of this condition, further investigation into its prevalence among women with PCOS, involving a larger sample, would be valuable. Our study did not establish a direct relationship between quality of life and the experience of having offspring or feelings of sadness. Instead, it revealed that lower quality of life was associated with women who acknowledged that PCOS was impacting their lives, women with a sense of lack of control over the disease, and women grappling with depression.

The treatment of PCOS is contingent upon the predominant symptoms and the individual patient’s expectations regarding therapeutic outcomes. Consequently, PCOS treatment should be comprehensive and tailored to the specific needs of each woman. In cases of insulin resistance and hyperinsulinemia, when nonpharmacological approaches like a proper diet and regular exercise prove insufficient, pharmacological treatment becomes necessary. Metformin, a widely utilized drug, is commonly prescribed. It enhances insulin sensitivity in target tissues and has a positive impact on the lipid profile. Metformin’s mechanism involves inhibiting hepatic gluconeogenesis, subsequently reducing intestinal glucose absorption, and later increasing peripheral glucose uptake and metabolism. Despite its side effects, metformin significantly improves tissue insulin sensitivity and contributes to weight loss. As an alternative to metformin, inositol supplementation is increasingly recommended [37,38,39,40,41]. Conversely, for women not planning to conceive but contending with irregular menstrual cycles and excessive hairiness, oral contraceptives are the preferred form of therapy. Their primary role is to regulate menstrual cycles and reduce hyperandrogenemia. Hormonal contraception inhibits excessive secretion of luteinizing hormone (LH) and lowers free androgens. It is crucial to note that the effects of hirsutism therapy may only become evident after 3–6 months due to the hair follicle development cycle. However, these effects may reverse upon discontinuation of treatment. Some women with PCOS are turning to cosmetic procedures like laser hair removal. Given the potential risk of embolic complications with oral contraceptives, it is essential to approach patients individually, selecting the safest contraception type while considering contraindications and assessing risk factors for heart disease [42,43,44,45,46]. Therefore, comprehensive care is vital, tailoring treatment based on accompanying symptoms, conditions, and expected outcomes. Early diagnosis and appropriately selected treatment can help prevent more serious consequences associated with untreated PCOS [1,27,29]. Unfortunately, in our study, we did not observe an impact of treatment on the assessed quality of life of women with PCOS.

## 5. Conclusions

Despite the majority of women with PCOS rating their quality of life as good or very good, they often find the associated symptoms of PCOS bothersome. Women reporting a lower quality of life tend to acknowledge the impact of PCOS on their lives, experience a sense of lack of control over the disease, struggle with depression, and do not accept their physical appearance. Hence, the support from specialists like endocrinologists, gynecologists, and nutritionists becomes crucial for many women dealing with PCOS. Adopting a healthy lifestyle, incorporating a balanced diet, and engaging in regular physical activity can assist in managing the troublesome symptoms of PCOS, thereby enhancing overall quality of life. In instances of emotional difficulties, seeking psychological support is equally important, and the significance of support and acceptance from loved ones should not be overlooked.

## Figures and Tables

**Table 1:** Characteristics of the study group.

Variable	Subgroup	%
Age	24–29 years	53%
	18–23 years	24.5%
	30–35 years	18.5%
	above 35 years	4%
Residence	large cities, >100 thousand residents	34.5%
	medium-sized cities, 50–100 thousand residents	15.5%
	small cities, <50 thousand residents	18%
	country	32%
Marital status	married	41%
	informal relationship	30.5%
	unmarried—maidens	27%
	widows	1%
	divorced	0.5%
Education	higher	60%
	secondary	34.5%
	vocational	3.5%
	primary	2%
Job status	active—working	71%
	studying	19.5%
	nonactive	9.5%
Socio-economic conditions	good	57%
	very good	30%
	sufficient	12%
	poor	1%

**Table 2:** Clinical symptoms and treatment options for polycystic ovary syndrome.

Variable	Subgroup	%
Diagnosis of PCOS	1–3 years	31.5%
	up to 1 year	24%
	4–6 years	22%
	up to 10 years	10%
	above 10 years	12.5%
Severity of symptoms	bothersome	40.5%
	moderately bothersome	26.5%
	very bothersome	24%
	little bothersome	8.5%
	nondisruptive	0.5%
Under constant care	gynecologist	96%
	endocrinologist	50.5%
	dietician	13%
	psychologist	11%
	dermatologist	8.5%
	psychiatrist	8%
	diabetologist	7.5%
Treatment undertaken	yes	79%
	no	21
Forms of treatment	hormonal contraception/yes	26%
	no	74%
	metformin/yes	39.5%
	no	60.5%
	diet/yes	53.5%
	no	46.5%
	physical activity/yes	51%
	no	49%

**Table 3:** Self-assessment of quality of life in women with polycystic ovary syndrome.

Variable	Subgroup	%
Self-assessment of quality of life	good quality of life	61.5%
	very good quality of life	15%
	poor quality of life	2.5%
	very bad quality of life	1%
Sadness	yes	79%
	no	21%
Depression	diagnosed	26%
	no depression	74%
Accept of their appearance	yes	40.5%
	no	59.5%
Self-esteem	low	67.5%
	not affected	32.5%
Influence on spheres of life	sexual sphere	79.2%
	family sphere	61.9%
	social sphere	50.6%
	professional sphere	24.4%

**Table 4:** Relationship between quality of life assessment, duration of illness, severity of symptoms, and comorbidities.

		How Is Your Quality of Life?			*χ* ^2^	*p*
		Very Good	Good	Sufficient/ Poor		
How long ago were you diagnosed with polycystic ovarian syndrome (PCOS)?	Less than a year ago	25.0%	58.3%	16.7%	8.98	0.344
	1–3 years ago	11.1%	73.0%	15.9%		
	4–6 years ago	22.7%	61.4%	15.9%		
	7–10 years ago	20.0%	50.0%	30.0%		
	11 or more years ago	28.0%	48.0%	24.0%		
How do you assess the annoyance of the symptoms present?	Little bothersome	33.3%	66.7%	0.0%	22.25	0.001
	Moderately bothersome	24.5%	69.8%	5.7%		
	Bothersome	21.0%	58.0%	21.0%		
	Very bothersome	8.3%	56.3%	35.4%		
Have you been diagnosed with any of the conditions that accompany PCOS?	One	22.9%	61.8%	15.3%	4.04	0.401
	Two	12.2%	65.4%	22.4%		
	More	21.2%	54.6%	24.2%		

*χ*^2^: Chi-square statistics; *p*: statistical significance level.

**Table 5:** Relationship between quality of life assessment, treatment, diet, and physical activity.

		How Is Your Quality of Life?			*χ* ^2^	*p*
		Very Good	Good	Sufficient/ Poor		
Are you being treated (for symptoms related to) polycystic ovary syndrome?	No	19.0%	57.2%	23.8%	1.00	0.607
	Yes	20.3%	62.6%	17.1%		
Do you use hormonal contraception?	No	23.0%	58.1%	18.9%	3.63	0.162
	Yes	11.5%	71.2%	17.3%		
Do you take metformin?	No	20.7%	61.1%	18.2%	0.09	0.956
	Yes	19.0%	62.0%	19.0%		
Do you follow any kind of diet?	No	14.0%	69.9%	16.1%	5.67	0.059
	Yes	25.2%	54.2%	20.6%		
Do you take care of regular physical activity (at least three times a week)?	No	16.3%	64.3%	19.4%	1.62	0.445
	Yes	23.5%	58.9%	17.6%		

*χ*^2^: Chi-square statistics; *p*: statistical significance level.

**Table 6:** Relationship between quality of life assessment, having children, and experiencing negative emotions.

		How Is Your Quality of Life?			*χ* ^2^	*p*
		Very Good	Good	Sufficient/ Poor		
Do you have any children?	No	18.3%	62.8%	18.9%	1.66	0.436
	Yes	27.8%	55.6%	16.8%		
Does the presence of PCOS affect any area of your life?	No	40.6%	56.3%	3.1%	13.14	0.001
	Yes	16.1%	62.5%	21.4%		
Do you experience anxiety/sadness due to PCOS?	No	26.2%	64.3%	9.5%	3.40	0.183
	Yes	18.4%	60.8%	20.8%		
Do you feel a lack of control over the situation with PCOS?	No	28.0%	68.0%	4.0%	10.17	0.006
	Yes	17.3%	59.4%	23.3%		
Do you have depression?	No	23.0%	65.5%	11.5%	19.16	0.000
	Yes	11.5%	50.0%	38.5%		

*χ*^2^: Chi-square statistics; *p*: statistical significance level.

**Table 7:** Relationship between quality of life assessment and self-esteem.

		How Is Your Quality of Life?			*χ* ^2^	*p*
		Very Good	Good	Sufficient/ Poor		
Do you accept your appearance (physical)?	No	13.5%	58.8%	27.7%	20.19	0.000
	Yes	29.7%	65.4%	4.9%		
Do you have low self-esteem due to polycystic ovary syndrome?	No	27.7%	63.1%	9.2%	7.36	0.025
	Yes	16.3%	60.7%	23.0%		
How do you assess the support from your family?	Very good	39.3%	53.6%	7.1%	22.13	0.001
	Good	21.9%	67.2%	10.9%		
	Moderate	15.1%	64.0%	20.9%		
	Poor	9.0%	45.5%	45.5%		

*χ*^2^: Chi-square statistics; *p*: statistical significance level.

## Data Availability

Data are available in a publicly accessible repository.
